# Sowing Silver Seeds within Patterned Ditches for Dendrite‐Free Lithium Metal Batteries

**DOI:** 10.1002/advs.202100684

**Published:** 2021-05-24

**Authors:** Hua Wang, Pei Hu, Xueting Liu, Yue Shen, Lixia Yuan, Zhen Li, Yunhui Huang

**Affiliations:** ^1^ State Key Laboratory of Material Processing and Die & Mold Technology School of Materials Science and Engineering Huazhong University of Science and Technology Wuhan 430074 China

**Keywords:** composite Li anodes, dendrite‐free, Li metal batteries, no gas generation, patterned ditches

## Abstract

The interfacial instability of lithium (Li) metal is one of the critical challenges, which hinders the application of rechargeable Li metal batteries (LMBs). Designing facile and effective surface/interface is extremely important for practical LMBs manufacturing. Here, a highly stable Li anode with silver nanowires sowed in the patterned ditches via a simple calendaring process is developed. The remarkably increased electroactive surface area and the superior lithiophilic Ag seeds enable Li stripping/plating mainly inside the ditches. Benefitting from such unique structural design, the ditches‐patterned and Ag‐modified composite Li anode (D‐Ag@Li) achieves excellent cyclability under 2 mA cm^−2^ / 4 mAh cm^−2^ over 360 h cycling with low nucleation overpotential of 16 mV. Pairing with the D‐Ag@Li anode, the full cells with LiNi_0.8_Mn_0.1_Co_0.1_O_2_ and LiFePO_4_ (LFP) cathodes achieve long cycle life with 94.2% retention after 2000 cycles and 74.2% after 4000 cycles, respectively. Moreover, ultrasonic transmission mapping shows no gas generation for the LFP pouch full cell pouch cell based on D‐Ag@Li over prolonged cycling, demonstrating the feasibility and effectiveness of the authors' strategy for LMBs.

## Graphical abstract

A highly stable lithium (Li) anode with silver nanowires (AgNWs) sowed in the patterned ditches is developed. The interconnected ditches significantly enhance the surface area of Li metal, and at the same time, AgNWs can serve as preferred electrochemical active sites to induce homogeneous Li nucleation and growth from the bottom rather than the top surfaces.

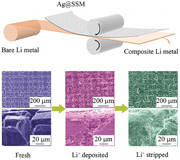

## 1 Introduction

Lithium (Li) metal anode has drawn much research attention due to its ultrahigh theoretical specific capacity (≈3860 mAh g^−1^), lowest redox potential (−3.04 V versus the standard hydrogen electrode), and low gravimetric density (0.534 g cm^−3^).^[^ ^1^, ^2^, ^3^, ^4^, ^5^, ^6^ ^]^ Nevertheless, the dendrite growth, infinite volume change, and high reactivity with the electrolyte of Li metal induce poor cycle life and severe safety problems, which strongly retard the commercialization of Li metal batteries (LMBs).^[^ ^7^, ^8^, ^9^, ^10^ ^]^ It is widely accepted that the Li dendrites' growth and volume expansion are caused by nonuniform Li nucleation and heterogeneous Li‐ion deposition. Recently, many strategies have been proposed to inhibit Li dendrites, such as selective Li nucleation,^[^ ^11^, ^12^, ^13^, ^14^, ^15^, ^16^, ^17^, ^18^ ^]^ seeded Li growth,^[^ ^19^, ^20^, ^21^, ^22^, ^23^ ^]^ host design,^[^ ^24^, ^25^, ^26^, ^27^, ^28^, ^29^, ^30^ ^]^ etc. According to Sand's time model, current density has significant effect on Li nucleation and growth.^[^ ^5^, ^6^ ^]^ Thus, increasing active surface area through 3D current collectors is thought to be an effective strategy to decrease local current density and inhibit Li dendrite growth. However, the loosened and porous structure of many 3D hosts will adsorb excess electrolyte and occupy larger electrode volume, which greatly decrease the gravimetric and volume energy densities of the composite Li metal anode. To address such concerns, some groups have directly constructed patterned ditches on the surface of bare Li foil to gain larger surface area for regulating the deposition behavior of Li.^[^ ^31^, ^32^, ^33^ ^]^ Although the nucleation overpotential and cycling stability can be improved, Li^+^ will preferably deposit on both tops and bottoms of the ditches under high current densities or high areal capacities, leading to Li dendrites growth over prolonged working time. Therefore, the above‐mentioned structures on Li surface still need to be further modified for more critical working conditions.

It is proven that, due to the high solubility in Li, gold (Au)^[^ ^15^, ^16^, ^17^ ^]^ and silver (Ag)^[^ ^21^, ^22^, ^23^, ^34^, ^35^, ^36^, ^37^, ^38^ ^]^ could effectively guide the nucleation and uniform growth of Li^+^. Some Au (Ag)/carbon composite nanostructures have been designed as hosts for Li metal anode, and obviously improved the cycle stability and Coulombic efficiency of LMBs. However, most of the previously reported Au or Ag‐based hosts are too complex and sophisticated to scale‐up for mass production.

Here, we develop a highly stable Li anode with silver nanowires (AgNWs) sowed within the patterned ditches by a simple calendaring process. The interconnected ditches have significantly enhanced the surface area of Li metal; at the same time, AgNWs inside the ditches can serve as preferred electrochemical active sites to induce homogeneous Li nucleation and growth from the bottom rather than the top of the patterned surfaces. In addition, the interconnected ditches with certain depth and width can also accommodate the huge volumetric change of Li during cycling. The obtained ditches‐patterned and silver‐modified composite Li anode (noted as D‐Ag@Li) exhibits high Coulombic efficiency, excellent cyclability, and prolonged cycle life in both symmetry and full cells.

## 2 Results and Discussion

**Figure 1:**
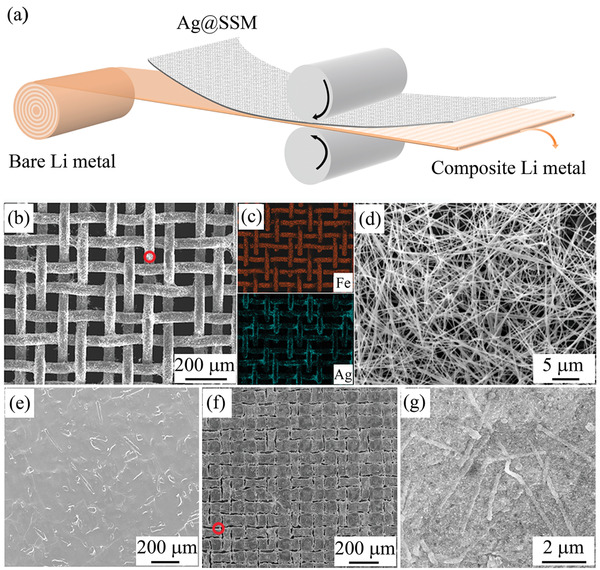
a) Schematic illustration of the fabrication process of the D‐Ag@Li composite. SEM images of b) SSM/Ag and d) AgNWs on the red circled surface of SSM. c) The corresponding EDX images of (b). SEM images of e) the bare Li foil, f) the D‐Ag@Li composite, and g) the high magnification of the red circled area in (f).

Li metal can be easily modified by physical treatment due to its soft and ductile properties. **Figure** **1**a illustrates the schematic of fabrication process of the patterned composite Li. Typically, the stainless steel mesh (SSM) with AgNWs attached on the surface is evenly placed on a bare Li foil and gently rolled by the polytetrafluoroethylene rods. The D‐Ag@Li composite can be obtained after removing the SSM. Figure 1b–d shows the scanning electron microscope (SEM) images and corresponding energy dispersive X‐ray (EDX) mapping of SSM with AgNWs dispersed on its surface, indicating that AgNWs are uniformly distributed and adsorbed on SSM. Compared with flat bare Li foil (Figure 1e), there are patterned and cross‐linked ditches on the surface of D‐Ag@Li (Figure 1f). The high‐magnification SEM image shows that AgNWs are well embedded on the bottom of the Li metal ditches (Figure 1g). The areal mass loading of AgNWs is around 0.07 mg cm^−2^, corresponding to 4.51 $ m^−2^, according to Table S1, Supporting Information, which would be 0.11 $ Ah^−1^ when the areal capacity of Li anode is set to 4 mAh cm^−2^. Such a simple process could be easily scaled up for mass production (Figure S1, Supporting Information).

**Figure 2:**
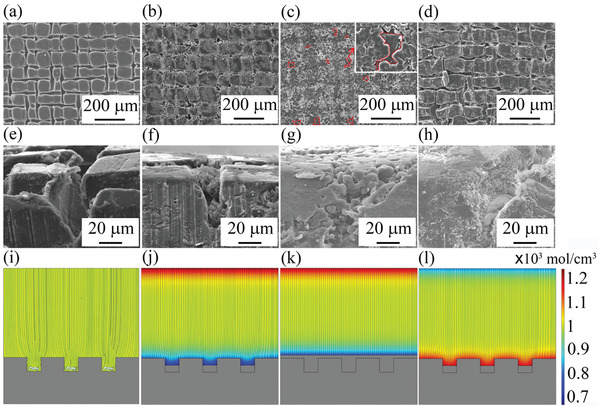
a–d) Top‐surface, e–h) cross‐section SEM images, and i–l) COMSOL simulation of D‐Ag@Li at (a,e,i) primary state, after (b,f,i) plating 0.5 mAh cm^−2^, (c,g,k) plating 2 mAh cm^−2^, and (d,h,l) Li stripping.

To confirm the plating/stripping behaviors of the as‐fabricated D‐Ag@Li composite, SEM observation and COMSOL simulation were conducted. **Figure** **2**a–e shows the SEM images of D‐Ag@Li with interconnected ditches before Li plating, and the electric field intensity and Li ion concentration are uniformly distributed on the anode surface at this stage (Figure 2i). When 0.5 mAh cm^−2^ of Li is plated, it is observed that most of Li is deposited inside the ditches (Figure 2b–f), indicating that AgNWs can effectively guide the Li deposition location, which is in good agreement with the COMSOL simulation (Figure 2j). When the deposition capacity increases to 2 mAh cm^−2^, it is found that Li would first fill the whole empty space of the ditches, and then grow on the top surfaces (Figure 2c,g–k). In the stripping process, it is predicted that the previously deposited Li can be more easily stripped from the surface instead of the original base Li metal (Figure 2l). SEM observation confirms that, after a Li stripping of 2 mAh cm^−2^, the D‐Ag@Li composite retains to its original structure with clear patterned ditches (Figure 2d–h).

**Figure 3:**
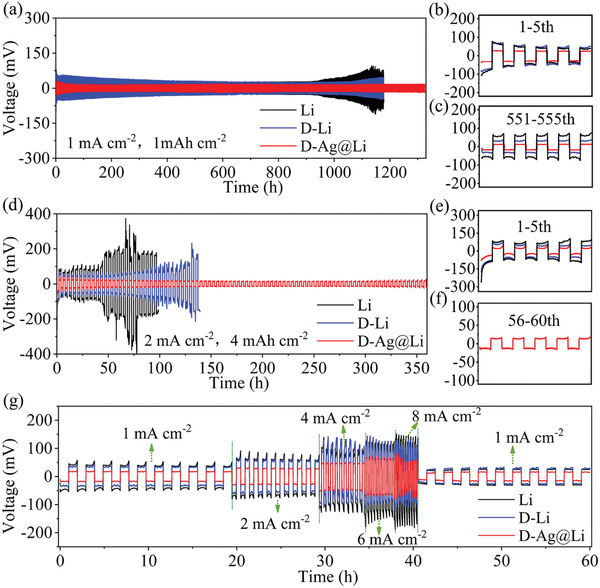
Galvanostatic cycling performance of D‐Ag@Li, D‐Li and bare Li anodes in symmetric cells at a) 1 for 1 mAh cm^−2^ and d) 2 for 4 mAh cm^−2^. b,c) and e,f) the corresponding voltage profiles of some cycles in (a) and (d), respectively. g) Rate performances of D‐Ag@Li, D‐Li and bare Li symmetric cells at various current densities for 1 mAh cm^−2^.

Symmetric cells with 50 µL ether‐based electrolyte were used to evaluate the electrochemical performance of various Li‐based anodes. The D‐Ag@Li symmetric cell cycled at 1 mA cm^−2^ for 1 mAh cm^−2^ displays extraordinary stability over 1350 h without fluctuation (**Figure** **3**a). And, it also maintains a polarization voltage of 13 mV, much lower than the cells based on D‐Li and bare Li anodes. More details of the galvanostatic voltage profiles for D‐Ag@Li electrode in Figure 3b,c further demonstrate more flat voltage plateau with a lower overpotential than that of the control electrodes. The calculated initial nucleation overpotentials of Li metal deposition are 3 (D‐Ag@Li), 20 (D‐Li), and 35 mV (bare Li) (Figure S2a, Supporting Information), indicating the effectiveness of D‐Ag@Li on lowering the nucleation barrier during Li deposition. When cycled at 2 mA cm^−2^ with areal capacity of 4 mAh cm^−2^, the D‐Ag@Li electrode still shows much better cycling performance (360 h) with stable polarization voltage and a low nucleation overpotential of 16 mV, whereas sharp oscillations occur after only 50 and 135 h cycles for bare Li and D‐Li electrodes, respectively (Figure 3d–f and Figure S2b, Supporting Information). To the best of our knowledge, bare Li metal anode has poor rate capabilities since a high current would accelerate the formation of Li dendrites. Figure 3g shows the rate performance comparison of D‐Ag@Li, D‐Li, and bare Li symmetric cells at various current densities for 1 mAh cm^−2^. The D‐Ag@Li composite anode presents a low polarization voltage of 58 mV at a high current density of 8 mA cm^−2^, much lower than those of the D‐Li electrode (85 mV) and bare Li electrode (145 mV).

**Figure 4:**
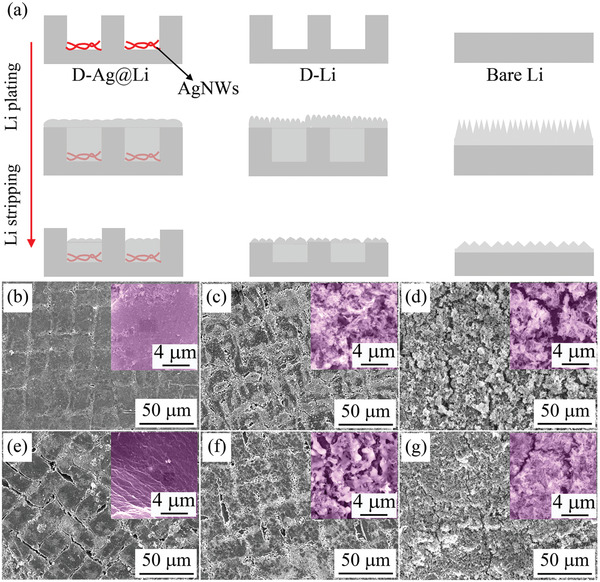
a) Schematic illustration of different Li depositing/striping behaviors of D‐Ag@Li D‐Li and bare Li. SEM images of b,e) D‐Ag@Li, c,f) D‐Li, and d,g) bare Li anodes after 10 cycles (1 mA cm^−2^ / 1 mAh cm^−2^) at b–d) plating and e–g) stripping status. The insets in (b–g) are the corresponding enlarged SEM images.

Different Li plating/striping behaviors of D‐Ag@Li, D‐Li, and bare Li are illustrated in **Figure** **4**a. SEM characterizations were further conducted to investigate the morphological changes of different anodes after 10 cycles at 1 mA cm^−2^ / 1 mAh cm^−2^ (Figure 4b–g). After 10 cycles at the Li fully deposited status, the newly deposited Li only stayed in the ditches of D‐Ag@Li, obtaining a very smooth surface (Figure 4b). After the same capacity of Li was stripped, the structure of the ditches recovered to a relatively intact status (Figure 4e). Without AgNWs in the ditches, the newly deposited Li filled a large part of the ditches for the D‐Li anode, but the subsequently plated Li formed dendrite morphology on its top surface (Figure 4c). When the Li was stripped, D‐Li showed a much rougher surface morphology than that of D‐Ag@Li (Figure 4f). As for the bare Li electrode, cracks and more serious dendrites could be clearly observed (Figure 4d,g). Even after 50 cycles, the D‐Ag@Li anode still maintained much smoother morphology and better array‐patterned structure than those of the D‐Li and bare Li anodes (Figures S3–S5, Supporting Information). The SEM observation results well confirm the excellent electrochemical reversibility of the D‐Ag@Li composite anode.

**Figure 5:**
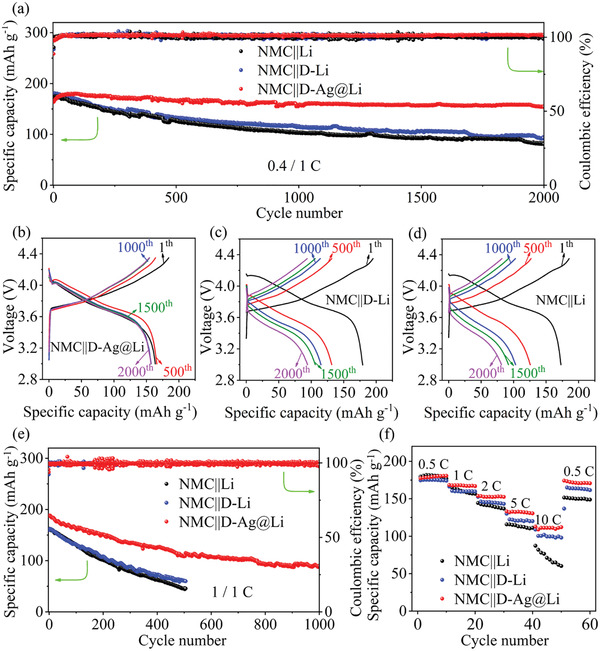
a) Cycling performance and b‐d) the corresponding voltage profiles of NMC||D‐Ag@Li, NMC||D‐Li, and NMC||Li full cells at 0.4/1 C charge/discharge condition. e) Cycling performance of the full cells at 1/1 C charge/discharge condition. f) Rate performance of the full cells at various current densities from 0.5 to 10 C.

To verify the effectiveness and feasibility of D‐Ag@Li in LMBs, full cells with LiNi_0.8_Mn_0.1_Co_0.1_O_2_ (NMC811) cathode were assembled and evaluated in carbonate‐based electrolyte at 0.4/1 C. For comparison, the D‐Li and bare Li anodes were tested under the same condition. The NMC||D‐Ag@Li full cell successfully delivers an initial discharge capacity around 156.6 mAh g^−1^, and maintains an ultralong cycle stability, retaining 94.2% of the initial discharge capacity even after 2000 cycles (**Figure** **5**a). Contrarily, the NMC||D‐Li and NMC||Li full cells only hold 51.7% and 46.7% capacity retention after 2000 cycles, respectively. This counterintuitive result is mainly attributed to the excellent interfacial stability of our arrays‐patterned Li electrode facilitated by large electroactive area. Moreover, the charge–discharge voltage profiles of the NMC||D‐Ag@Li full cell show much lower polarization than the NMC||D‐Li and NMC||Li cells (Figure 5b–d), indicating that D‐Ag@Li has a much better interfacial stability during cycling test.

High rate charge–discharge cycling is common in practical conditions, but is very critical for LMBs, since the high areal current density will accelerate Li dendrite growth. Here, to show the excellent stability of the proposed D‐Ag@Li, a relatively high charge rate of 1 C was applied on the full cells for cycling tests. As shown in Figure 5e, the full cell based on D‐Ag@Li shows prolonged cycling stability with discharge capacity up to 89.3 mAh g^−1^ after 1000 cycles. On the contrary, the capacities of the D‐Li and Li‐based full cells decreased to 45.7 and 60.7 mAh g^−1^ after 500 cycles, respectively. These results demonstrate that the surface of D‐Ag@Li composite anode can withstand fast charge/discharge cycling. Rate performances of NMC||D‐Ag@Li, NMC||D‐Li, and NMC||Li full cells were also investigated at 0.5, 1, 2, 5, and 10 C (Figure 5f). NMC||D‐Ag@Li full cell exhibits superior rate capacities up to 132.1 and 111.1 mAh g^−1^ even under 5 and 10 C, respectively. Finally, in order to reveal the competitive advantages of D‐Ag@Li anode in different LMBs, LiFePO_4_ (LFP) cathode‐based full cells were further constructed and tested at 5 C (Figure S6, Supporting Information). The LFP||D‐Ag@Li full cell show a capacity retention of 74.2% with low voltage polarization after 4000 cycles, which is much better than those of LFP||D‐Li and LFP||Li. Even at high rate of 10 C, it can still show excellent cycle stability over 3200 cycles with an average capacity decay of 0.0045% per cycle (Figure S7, Supporting Information). Moreover, as shown in Figure S8, Supporting Information, the full cell using D‐Ag@Li anode also shows a much better rate performance than the cells using D‐Li and Li anodes. At the same time, it maintains a significantly lower overpotential at higher rates of 10 and 20 C.

**Figure 6:**
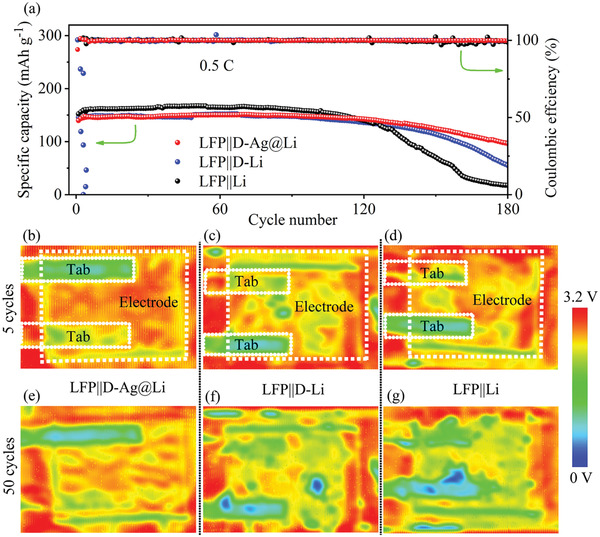
a) Cycling performance of LFP||D‐Ag@Li, LFP||D‐Li, and LFP||Li pouch cells at 0.5 C. b–g) Ultrasonic transmission mapping of the three pouch cells after b–d) 5 cycles and e–g) 50 cycles.

We further explored the advantages of the D‐Ag@Li in more practical cell models. Three kinds of LFP||D‐Ag@Li, LFP||D‐Li, and LFP||Li single‐layer pouch cells with LFP mass loading of 200–240 mg (3 × 4 cm^−2^) were assembled. As shown in **Figure** **6**a, the LFP||D‐Ag@Li pouch cell delivers a high capacity of 19.8 mAh even after 180 cycles, while it is 11.8 and only 4.2 mAh for the D‐Li and bare Li counterparts, respectively. Since other parameters are kept the same, the desirable electrochemical performance should be attributed to the excellent interfacial stability of the designed D‐Ag@Li composite anode. Furthermore, ultrasonic transmission mapping,^[^ ^39^, ^40^ ^]^ a very sensitive technique, was performed to detect the gas generation process inside the pouch cells. Due to the difference of ultrasonic transmissivity in different medias (*T*_gas_> *T*_liquid_ > *T*_solid_), the transmitted ultrasonic wave is seriously reflected or scattered at the gas–liquid and gas–solid interface, leading to severe transmission signal attenuation. In monitoring gas generation for pouch cell, the collected ultrasonic signals at each point are converted into a red‐to‐blue heat map. The blue color represents the low ultrasonic transmittance, indicating serious gas generation caused by bad side reactions including electrolyte decomposition and the repeated break and repair of SEI during cycles. The red color represents strong ultrasonic signals, demonstrating superior interfacial contact. Figure 6b–g shows the ultrasonic transmission images of three pouch cells after 5 and 50 cycles. It can be seen clearly that there is no obvious color change for the LFP||D‐Ag@Li pouch cell at 5 and 50 cycles status. However, it became green and blue after 50 cycles for the LFP||D‐Li and LFP||Li pouch cells, indicating serious interfacial side reaction and gas generation. Therefore, D‐Ag@Li shows its potential for gaining a much enhanced high safety in commercial LMBs.

## 3 Conclusion

In summary, a novel arrays‐patterned composite Li anode has been rationally designed to mitigate (Li) dendrite growth. AgNW is introduced to regulate electric field to guide controllable plating and stripping of Li in and out of Li ditches due to its superior lithophilic nature. Moreover, the massive interconnected ditches effectively increase electroactive surface area, which is favorable for locally reducing current density. Benefitting from the structure and material uniqueness, the D‐Ag@Li anode shows excellent performances in various battery systems. The D‐Ag@Li composite anode also affords improved performance in a pouch cell with no gas generation. We believe that the present work not only provides a relatively facile and effective strategy for improving the electrochemical performance of Li metal anodes, but also has a potential to inspire the design of other metal anodes in various energy storage systems.

## 4 Experimental Section

### Preparation of AgNWs

Silver nitrate (AgNO_3_), ethylene glycol (EG), iron (III) chloride hexahydrate (FeCl_3_·6H_2_O), and copper (II) chloride dihydrate (CuCl_2_·2H_2_O) were purchased from Sinopharm Chemical Reagent Co. Ltd. Polyvinyl pyrrolidone (PVP, K90) was purchased from Shanghai Aladdin Biochemical Technology Co., Ltd. All chemicals were used without further purification. In a typical process, EG solution of PVP‐K90 (0.15 m, 10 mL), CuCl_2_·2H_2_O (0.6 mm, 0.75 mL), FeCl_3_·6H_2_O (0.6 mm, 0.75 mL), and AgNO_3_ (0.1 m, 5 mL) were first mixed in a glass beaker and vigorously stirred for 1 min, and the obtained uniform mixed solution was transferred into a 50 mL Teflon‐lined autoclave container, which was then sealed and maintained at 140 °C in an oven for 4 h. Finally, the products were obtained after centrifuging and then dried at 60 °C. The total mass of AgNWs is 48.6 mg with 90% productivity.

### Preparation of Ag@SSM Substrate

Ag@SSM substrate was prepared by a simple dipping method. In a typical process, a 300 mesh bulk SSM was cut into square pieces with an edge length of 15 mm, and AgNWs ink was prepared by dispersing the as‐synthesized AgNWs powder in ethanol at the concentration of 1 mg mL^−1^. The square SSM was then immersed into 500 µL AgNWs ink completely for 10 s, and finally, the Ag@SSW substrate was obtained after drying at 60 °C for 0.5 h.

### Preparation of D‐Ag@Li

The D‐Ag@Li composite was prepared by a calendaring process. Typically, the as‐prepared Ag@SSM was put on a fresh Li strip (110 µm) and rolled with Teflon rod. After removing the SSM substrate, the D‐Ag@Li composite was rinsed gently with dimethoxymethane solvent to remove the AgNWs that fell on the top of patterned surfaces and punched into the circular piece with diameter of 12 mm and thickness of 101 µm. D‐Li anode was prepared by the same method but with bare SSM.

### Characterization

The morphology and structure of the samples were characterized by field‐emission scanning electron microscopy (FESEM, Sirion 200). Energy‐dispersive X‐ray spectroscopy attached to the Sirion 200 was used to conduct the elemental analysis of Ag@SSM substrate. The observed composite Li anode samples were attained after plating or stripping a definite amount of Li and were rinsed gently by dimethoxymethane solvent to remove the residual electrolyte and LiTFSI salt.

### Electrochemical Measurements

The electrochemical performance was measured by using CR2032‐type coin cells assembled in an Ar‐filled glove box with O_2_ and H_2_O contents less than 1 ppm. The separator was Celgard 2300 membrane and the electrolyte was 1 m lithium bis(trifluoromethanesulfonyl)imide (LiTFSI) in 1,3‐dioxolane (DOL) and dimethoxymethane (DME) in a volume ratio of 1:1 with 2% LiNO_3_ additives. To standardize, a 50 µL electrolyte was used in each coin cell. For a coin‐type full cell and pouch cell measurements, the cathode was prepared by casting NMC811 or LFP slurry onto a carbon‐coated aluminum foil. The slurry was composed of commercial NCM811 (or LFP) powder, carbon black, and polyvinylidene fluoride (PVDF) binder (8:1:1 by mass) in *N*‐methyl‐2‐pyrrolidone (NMP). The NMC811 and LFP mass loadings were controlled at 1.5–3.5 mg cm^−2^ for coin full cells, and the LFP mass loading was about 200–240 mg for pouch cells with a size of 3 × 4 cm^−2^. The electrolyte was 1 m lithium hexafluorophosphate (LiPF_6_) with 5% fluoroethylene carbonate (FEC) additive in ethylene carbonate (EC) and diethyl carbonate (DEC) in a volume ratio of 1:1 (carbonate‐based electrolyte system). For a fair comparison, 50 and 500 µL electrolytes were used in each coin cell and pouch cell, respectively. The galvanostatic charge/discharge measurements of the full cells were carried out in a voltage cut‐off window of 4–2.2 V based on LFP electrodes and 4.35–3 V for NMC811 electrodes. All the galvanostatic charge/discharge experiments were conducted on a LAND testing system (Wuhan China). The morphology and structure of the samples were characterized by field‐emission scanning electron microscopy (FESEM, Sirion 200). Energy‐dispersive X‐ray spectroscopy attached to the Sirion 200 was used to conduct the elemental analysis of Ag@SSM substrate. The observed composite Li anode samples were attained after plating or stripping a definite amount of Li and rinsing gently using a DME solvent to remove the residual electrolyte and LiTFSI salt.

## Conflict of Interest

The authors declare no conflict of interest.

## Supporting information

Supporting Information

## Data Availability

Research data are not shared.
