# Supplementary material for: Sowing Silver Seeds within Patterned Ditches for Dendrite‐Free Lithium Metal Batteries
Source: Adv Sci (Weinh). 2021 May 24;8(14):2100684. doi: 10.1002/advs.202100684 (PMC8292901; doi:10.1002/advs.202100684)
Supplement: Supplementary file 1 — Supporting Information [file ADVS-8-2100684-s001.pdf]

WILEY-VCH

© 2021 Wiley-VCH GmbH

ADVANCED SCIENCE
Open Access

Supporting Information

for *Adv. Sci.*, DOI: 10.1002/advs.202100684

Sowing Silver Seeds within Patterned Ditches for Dendrite-free Lithium Metal Batteries

Hua Wang, Pei Hu, Xueting Liu, Yue Shen, Lixia Yuan, Zhen Li and Yunhui Huang**

Supporting information

Sowing Silver Seeds within Patterned Ditches for Dendrite-free Lithium Metal Batteries

Hua Wang, Pei Hu, Xueting Liu, Yue Shen, Lixia Yuan, Zhen Li and Yunhui Huang**

Dr. H. Wang, Dr. P. Hu, X. T. Liu, Dr. Y. Shen, Prof. L. X. Yuan, Prof. Z. Li, Prof. Y. Huang
State Key Laboratory of Material Processing and Die & Mold Technology
School of Materials Science and Engineering
Huazhong University of Science and Technology
Wuhan 430074, China
E-mail: li_zhen@hust.edu.cn; huangyh@hust.edu.cn

COMSOL simulation details: The distributions of electric and Li ions concentration were simulated through COMSOL Multiphysics without considering possible side effects. The whole simulation is a transient model during both Li plating and stripping processes, and which was defined by the mass conservation and electronegativity conditions of the related ions (Li^+ and electrolyte anion m^-). According to Nernst-Planck, the mass conservation equation is:

$$\frac{\partial c_i}{\partial t} + \nabla \cdot N_i = 0$$

Where N_i is the transfer vector (mol/(m^2·s)), c_i is the electrolyte concentration (mol/m^3), and the electronegativity condition is expressed by the following expression:

$$\sum_i z_i c_i = 0$$

Where z_i is ionic charge number. The chemical equivalent coefficients for Li ions in electrolyte and Li atoms on electrodes both were set as 1.

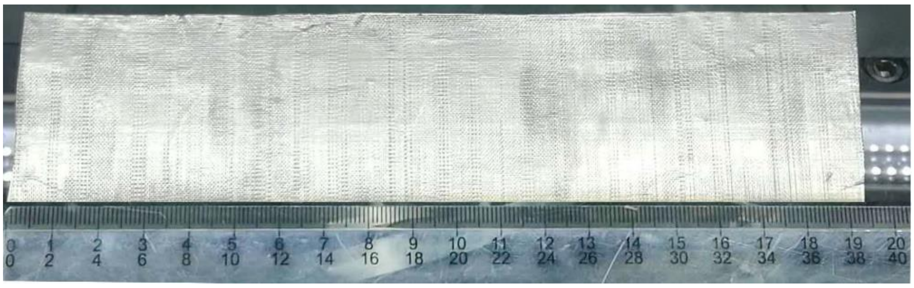

Figure S1. (a) Digital photo of the scaled-up preparation of D-Ag@Li.

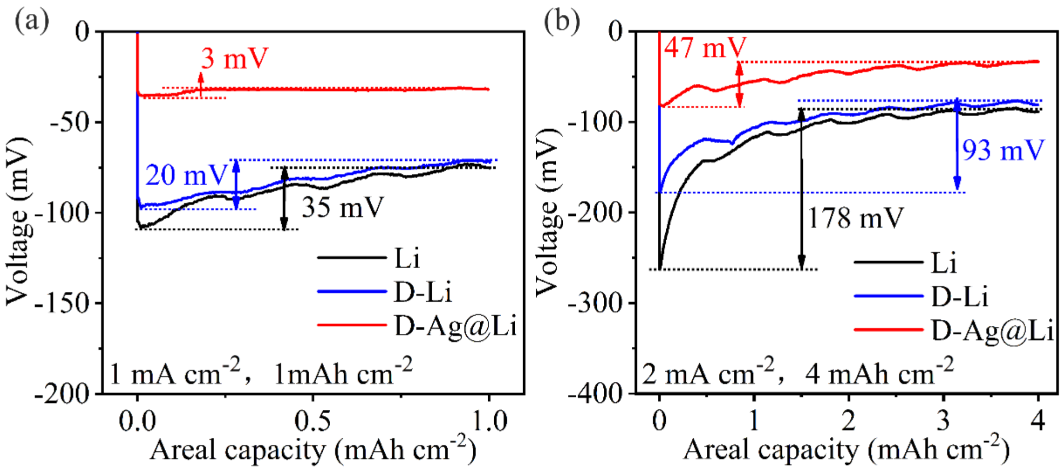

Figure S2. The nucleation overpotentials of D-Ag@Li, D-Li and Li symmetric cells at (a) 1 mA cm^{-2}/1 mAh cm^{-2} and (b) 2 mA cm^{-2}/4 mAh cm^{-2}

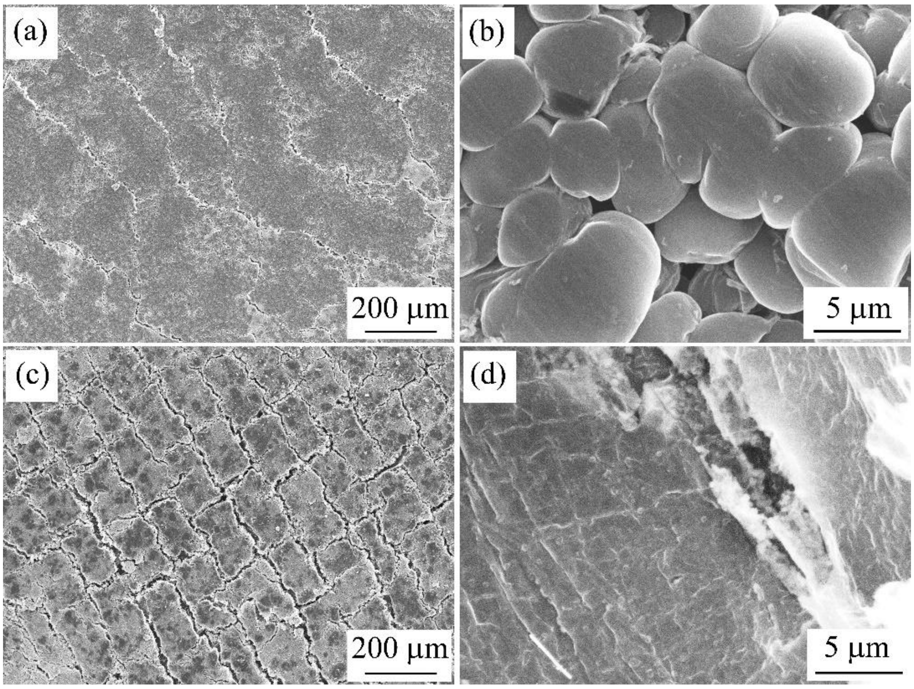

Figure S3. SEM images of D-Ag@Li after 50 cycles (1 mA cm^{-2} / 1 mAh cm^{-2}) at (a, b) plating and (c, d) stripping status.

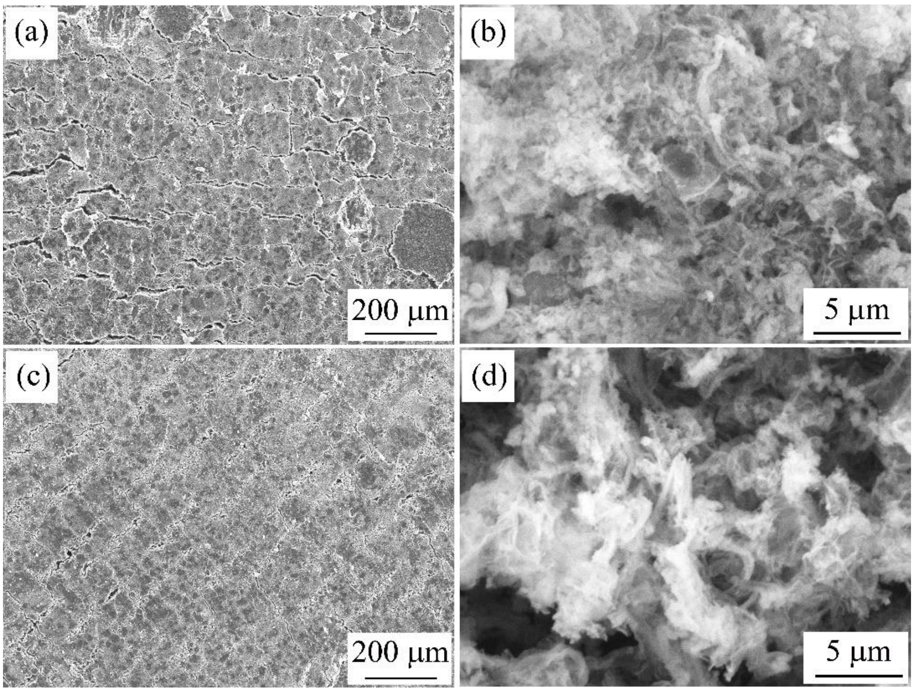

Figure S4. SEM images of D-Li after 50 cycles (1 mA cm^{-2} / 1 mAh cm^{-2}) at (a, b) plating and (c, d) stripping status.

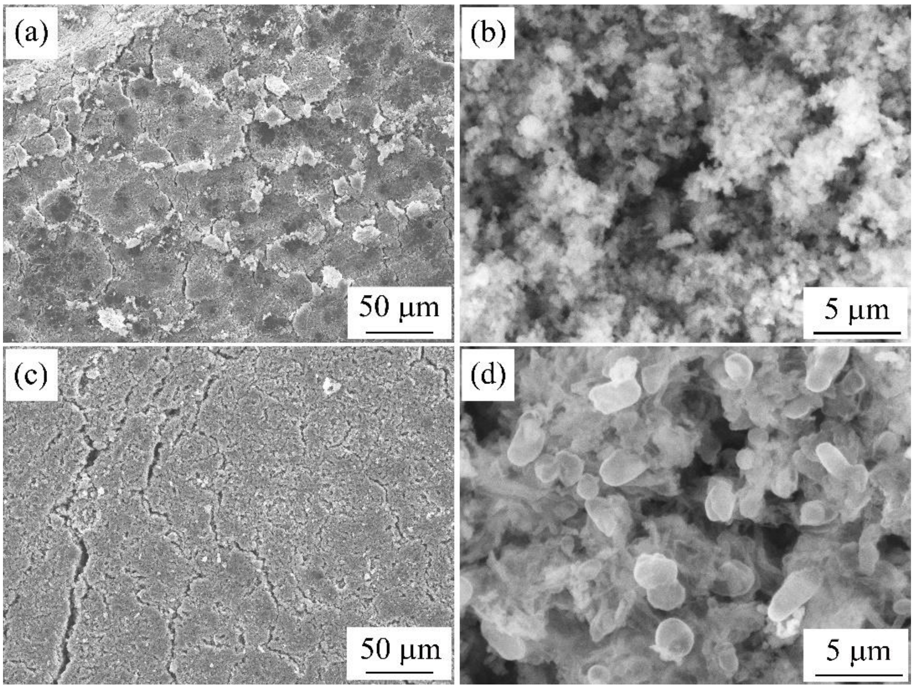

Figure S5. SEM images of bare Li after 50 cycles (1 mA cm^{-2} / 1 mAh cm^{-2}) at (a, b) plating and (c, d) stripping status.

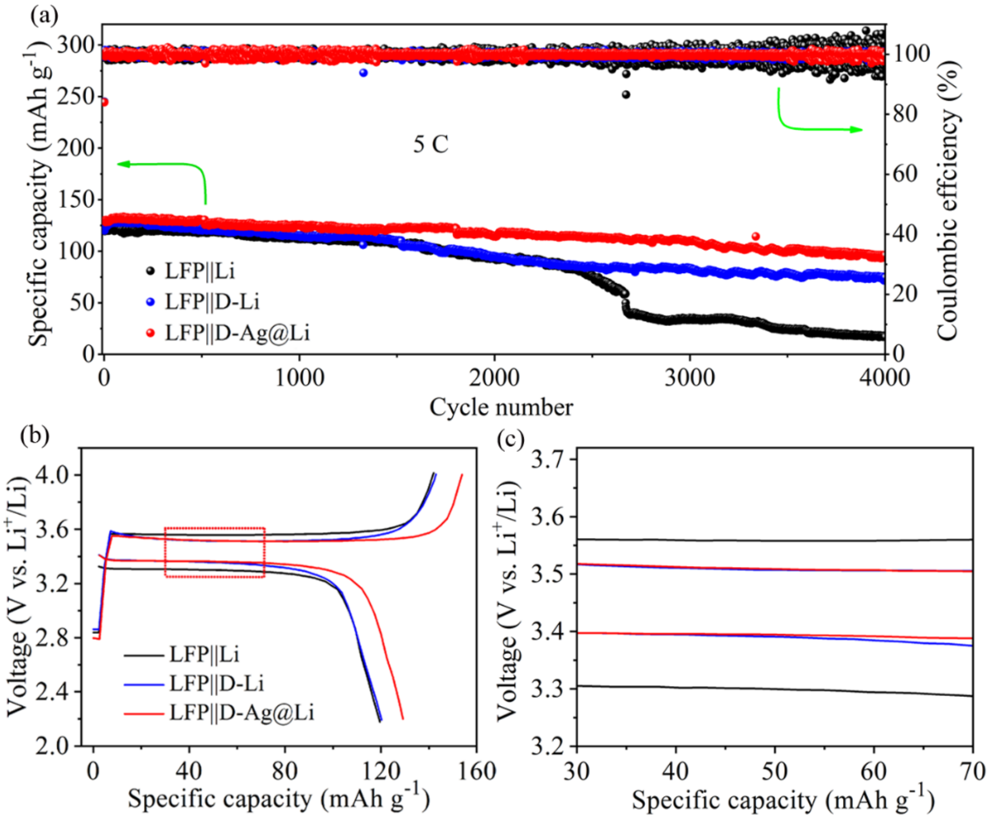

Figure S6. (a) Cycling performance of LFP||D-Ag@Li, LFP ||D-Li and LFP||Li full cells at 5 C. (b) The corresponding voltage profiles for the three full cells for the 1st cycle. (c) The enlarged image of the red square area in (b).

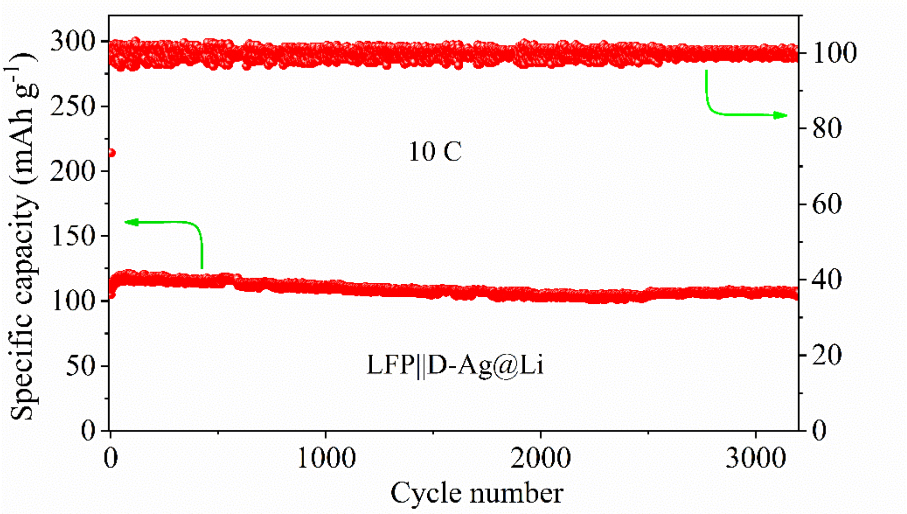

Figure S7. Cycling performance of LFP||D-Ag@Li at high current density of 10 C.

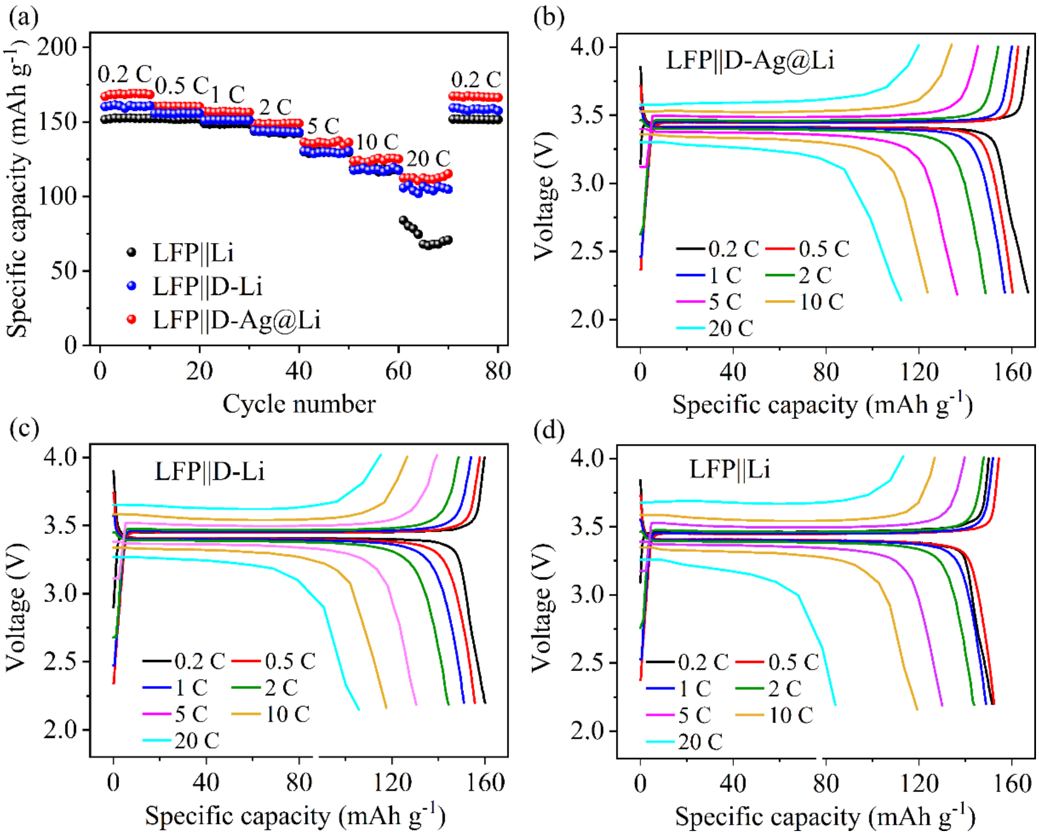

Figure S8. (a) Rate performance and the corresponding charge/discharge curves of (b) LFP||D-Ag@Li, (c) LFP ||D-Li and (d) LFP||Li full cells at various current densities.

Table R1. The cost analysis of manufacturing 1 g of AgNWs.

Reagent	Specification	Price ($)	Amount used	Cost ($)
**$AgNO_3$**	25 g	39.4	1.749 g	~2.756
**EG**	500 mL	5.078	339.5 mL	~3.448
**PVP-K90**	500 g	33.087	3.426 g	~0.227
**$CuCl_2 \cdot 2H_2O$**	500 g	11.542	1.584 mg	~0.000037
**$FeCl_3 \cdot 6H_2O$**	500 g	4.925	2.51 mg	~0.000025
**Total cost**				~6.431$ g^{-1}
